# Masks, Mingling and Magic: Gibberish Law in the Age of Covid

**DOI:** 10.1007/s10991-021-09288-x

**Published:** 2021-10-26

**Authors:** Ian Ward

**Affiliations:** Newcastle Law School, Windsor Terrace, Newcastle University, Newcastle upon Tyne, UK

**Keywords:** Covid-19, Percy Shelley, Edward Colston, Public Health Act, Carl Schmitt

## Abstract

The experience of Covid-19 has taught us many things, not least the consequence of what John Milton termed ‘gibberish law’. Law drafted amidst the ‘throng and noises of irrational men’. The closer purpose of this article is the attempt to regulate ‘gatherings’ during the coronavirus pandemic, including the re-invention of a bespoke crime of ‘mingling’. A jurisprudential curiosity which, it will be suggested, is symptomatic of a broader malaise. An assault on the integrity of the rule of law which is only too familiar; much, it might be said, like the arrival of a pandemic. The first part of the article will revisit three particular gatherings, in part to debunk the myth of the unprecedented. But also to introduce some themes, literal and figurative, of masking and muddle. The conjuring of what Shakespeare called ‘rough magic’. The second part of the article will then take a closer look at the jurisprudential consequence of this conjuration. The final part will venture some larger concerns, about the crisis of parliamentary democracy in the ‘age of Covid’.

The experience of Covid-19 has taught us many things, not least the consequence of what John Milton termed ‘gibberish law’. Law drafted amidst the ‘throng and noises of irrational men’.^1^ The closer purpose of this article is the attempt to regulate ‘gatherings’ during the coronavirus pandemic, including the re-invention of a bespoke crime of ‘mingling’.^2^ A jurisprudential curiosity which, it will be suggested, is symptomatic of a broader malaise. An assault on the integrity of the rule of law which is only too familiar; much, it might be said, like the arrival of a pandemic.^3^ The first part of the article will revisit three particular gatherings, in part to debunk the myth of the unprecedented. But also to introduce some themes, literal and figurative, of masking and muddle. The conjuring of what Shakespeare called ‘rough magic’.^4^ The second part of the article will then take a closer look at the jurisprudential consequence of this conjuration. The final part will venture some larger concerns, about the crisis of parliamentary democracy in the ‘age of Covid’.

## Gatherings

As advertised, we will revisit three gatherings, stretching across two centuries, and taking us from Manchester, via northern Italy, to south London, and then Bristol. Our purpose, again, is to spot some resonances, and introduce some themes, prosaic and poetic, each of which speaks to the consequence of ‘gibberish law’.

### An Afternoon in Manchester

Starting then in summer 1819, on the Ligurian coast, at the Villa Volsovano. The home, in that moment, of the poet Percy Shelley. Shrouded in ‘melancholy’, having just buried his youngest child, his wife Mary enduring another bout of manic depression, his consolation was a large glass tower, to which he could escape, to stare over the bay, and read the newspapers, when he had the energy.^5^ Mary spoke of a ‘listless’ moment. The papers took around two weeks to arrive from London. So it was not until 5th September that he read account of a gathering some at St Peter’s Fields in Manchester, which had taken place nineteen days earlier.

Around 60,000 had turned up to listen to the famed Henry ‘Orator’ Hunt; arguing the case for franchise reform. Many from miles around, marching in organized groups, with bands and banners, carrying mottoes such as ‘Liberty and Fraternity’ and ‘Unity and Strength’.^6^ Affronted by the presence of the rioters, fearing that the situation might run out of control, the magistrates had ordered the Riot Act to be read. A handful might have heard it, not many. No-one moved. So the magistrates sent in the yeomanry to disperse the crowds. To no avail. At which point they ordered in six troops of Hussars, waiting outside the ‘fields’. A few minutes later, eleven lay dead, hundreds injured.^7^ The press dubbed it the ‘Peterloo massacre’. Many of the Hussars had fought at Waterloo, and wore their battle-medals as they charged into the crowd. Not so much to be proud of here, the insinuated ran, hacking down unarmed women and children.

The day after reading the report, Shelley wrote to his friend, Thomas Love Peacock, attesting to the ‘torrent of indignation… not yet done boiling in my veins’. He waited ‘anxiously to hear how the Country will express its sense of this bloody murderous oppression of its destroyers’. The ‘dread lighting’ of vengeance.^8^ Meantime he would write one of the greatest poems of political protest in the English language.^9^ He called it *The Mask of Anarchy*. Ninety-one stanzas, in three sections. The first thirty-four tore into the government of Lord Liverpool. A series of caricatures, as would be written into a Court masque. Murder, Fraud, Hypocrisy; attaching to particular political figures. Here, respectively, Castlereagh, Eldon and Sidmouth. Others too of course. But these were the three who Shelley held especially responsible. Eldon was the Lord Chancellor, with whom Shelley anyway held a grudge.^10^ Castlereagh was Foreign Secretary, the dominant figure in the Cabinet. Sidmouth was the Home Secretary, who had sent instruction to the Manchester magistrates. It is they who ride into the crowd at St Peter’s Field.

The poem reaches its brilliant crescendo in the ninth stanza, with the arrival of Anarchy; the figure before which all the ministerial sycophants cower:

Last came Anarchy: he rode

On a white horse, splashed with blood;

He was pale even to the lips,

Like Death in the Apocalypse.

…

And he wore a kingly crown;

And in his grasp a sceptre shone;

On his brow this mark I saw –

I AM GOD AND KING AND LAW! (30-7)^11^

Plain enough. But there are twists. Not least in the stanza which immediately follows:

Over the English land he passed,

Trampling to a mire of blood

The adoring multitude. (39-41)

We will return to this ‘adoring’ later. It insinuates something about how anarchy is sustained. But also by how it might, ultimately, be defeated. The subject, indeed, of the second set of stanzas. Anarchy overcome by Hope. In the figure of the ‘maniac maid’ who stands up to the onslaught; an image which Shelley took from the account of a young woman trampled to death by the Hussars.

The third set of stanzas paints a brighter picture, of democracy and liberty, in a distinctly English hue:

The old laws of England – they

Whose reverend heads with age are gray,

Children of a wiser day;

And whose solemn voice must be

Thine own echo – Liberty! (333-5)

At the title supposes, Shelley wants us to think about anarchy and masks, and how the latter covers the former. We will start with the anarchy, which meant something different then. Shelley knew all about the politics, and the theatre, of tyranny. He had written about it in his epic poem on the French Revolution, *The Revolt of Islam*, and plenty more sonnets and verses.^12^ He had read Edmund Burke too, and imbibed the idea that ‘terror’ is the principle weapon with which the modern state assures itself. By 1819, the ‘aesthetics of terror’ was the defining presence in Shelley’s writing.^13^ His mind turned by William Godwin’s *Enquiry Concerning Political Justice*, at the heart of which was a simple thesis.^14^ Reversing Hobbes’s dour prediction, Godwin supposed that the ultimate state of political Enlightenment was liberty. Shelley writes this inversion into the heart of his *Mask*. Anarchy is not found in the crowds that protest, but is rooted in the terror of those who fear the protest. Which in August 1819 was the Manchester magistrates, and their masters in London. It was the ‘tyrants’ sat around Liverpool’s Cabinet table who had ‘first shed blood’.^15^

There are two reasons why Shelley’s poem has a particular prescience. The first is prosaic, the familiar truth, that governments, trapped and themselves terrified, tend to react violently. And they think nothing of cutting down liberty as they go. The second is to spot the more poetic resonances. Two again. First, that Shelley wrote a protest poem; about a protest. And second, the central metaphorical conceit; the mask and the masque. The wearing of a mask in order to prevent viral transmission during a pandemic may have a prosaic purpose. But it also has a semiotic resonance. In its original, tightly-conceived, design, the Renaissance ‘masque’ was all about the restoration of order. A first part depicting the ‘state of nature’, and its consequence, and a second during which calm is restored by a wise magistrate; albeit with the turn of a neatly placed heel, rather than the flash of a sabre-blade.^16^ Or the risk of a spot-fine.

Shelley knew all of this. It is why he selected the metaphor. The spectacle of government, purposed to awe. A masque of terror. With, in the end, a simple enough message: strip away the mask, and beneath lies the familiar face of tyranny. He closed optimistically:

Shake your chains to earth like dew

Which in sleep had fallen on you –

Ye are many – they are few.

An excited, and exhausted, Shelley despatched a finalised draft of the *Mask* to his friend Leigh Hunt, editor of the *Examiner*, at the end of the month. The news, when it finally filtered back, was disappointing. Threatened with prosecution for sedition, Hunt could not publish.^17^ The wrong moment.^18^

### An Evening on Clapham Common

At which point we will move forward two centuries, to 13th March 2021. Three days earlier the body of a missing woman named Sarah Everard had been discovered. Murdered, it transpired, by an off-duty police officer. The last sighting of Sarah had been near Clapham Common. An organisation named ‘Reclaim These Streets’ (RTS) approached the Metropolitan Police in order to facilitate a peaceful vigil, in accordance with existing Covid ‘regulations’. Which should have been possible, but proved not to be. The vigil was duly cancelled, but people continued to turn up during the day to pay their respects, many laying wreaths near a band-stand. As the evening drew on, numbers increased. Until, at some point, a fateful decision was made by senior police officers. Informed that the gathering was ‘static’ and ‘growing’, and might conceivably ‘present a health risk’, it was decided that the ‘vigil’ had transmuted into a ‘protest’, and should be dispersed. A dispersal order was read out. A handful might have heard it, not many. No-one moved. Absent any hussars, police snatch-squads were sent in; in the process radically increasing the risk of viral transmission, and setting in train what would prove to be a marketing disaster.^19^

The Home Secretary asked the Inspectorate of Constabulary to provide a ‘bespoke thematic’ report. Which returned a largely exculpatory verdict a few weeks later, albeit with a couple of caveats. Both reinforcing concerns expressed by the Metropolitan Police. The first bemoaned the marketing disaster, the second the state of the ‘frequently changing’ law. The matter did not, however, rest. Two months later, an All-Party Parliamentary Group (APPG) on ‘Democracy and the Constitution’ reviewed the events at Clapham Common. It concluded that the Metropolitan Police had failed on various accounts. First to engage ‘constructively’ with RTS. Second to ‘give transparency and clarity’ as to how it intended to police the regulations. Third, in failing to ‘conduct a proper assessment of the proportionality’ of its subsequent response to the ‘verbal resistance’. Such were the operational failures. Perhaps more serious were a set of broader intimations. First that the Met had applied a ‘presumption of illegality’ that was wrong in law. Something that was emphasised by Holgate J in his ruling, the day before the planned vigil, in *Leigh *et al*. v Commissioner for Metropolitan Police*.^20^ Second, that it failed to appreciate that the right to protest should be ‘supported, and not suppressed’ by the law. Third, that it had acted in a way that more closely resembled an ‘enforcement agency of the state’.^21^ Each intimation, it might be said, a little more troubling than the last.

The Everard vigil will continue to stimulate debate for some time. As it should. We will shortly contemplate the extent to which the events which unfolded on 13th March might be ascribed to the hazards of ‘gibberish’ law. In the meantime, though, we will contemplate the related issue, the adverse impact of the imagery. It matters every bit as much. Mary Shelley later recalled her husband roused to ‘indignation and compassion’ by what he had read of the events at St Peter’s Field.^22^ Less need of newspapers today, or indeed poetic testament. Twitter trades on indignation. Images of burly male officers flinging slightly built women to the floor were flashing round with world within minutes. Long before the Home Secretary could express her dismay, the following morning, at how easily such ‘images’ might be ‘taken out of context’.^23^

Dismayed perhaps, but there was certainly no reason to be surprised. At least not, if the Home Secretary had read her Shelley. Or indeed her Bagehot. A journalist by training, Walter Bagehot knew all about the darker arts of public perception. How to spot a ‘political cheapjack’, how to play the part, how, most importantly, to keep the ‘bovine’ masses in a state of ‘mystic reverence’.^24^ It was for this reason that he put so much stock in the ‘charmed spectacle’ of the monarchy, to mask the enormous powers exercised by the ‘secret prerogative’ of the Crown.^25^ His near-contemporary Albert Venn Dicey would try to console himself with the thought that certain principles, such as the rule of law, might temper an over-mighty executive. Bagehot barely bothered to hide his disdain; not just in the rule of law, but in attendant ideas of parliamentary sovereignty, or separate powers, checked and balanced.^26^ The reason is simple enough. History revealed, time and again, that government is as much a matter of sorcery as it is of sense. A kind of ‘magic’, indeed, upon which ‘daylight’ must never be shed.^27^

Later in the essay, we will contemplate John Austin’s suggestion, that the facility of law depends, ultimately, on a ‘habit of obedience’.^28^ It is, in a sense, a variant on the theme. Except that where Austin believed that reason and clarity were the key, Bagehot thought the converse. Either way, it matters, never more so than during a crisis, when maintaining public support is so critical. Regardless of the ‘legalities’ of the policing response at Clapham Common on 13th, no-one can conclude that it was anything but a marketing disaster. And not, sadly, the only such instance of ill-judged policing evinced over the last year and a half; chief Constables threatening to peer into supermarket trolleys to check if items for purchase were really ‘essential’; tweets reminding prospective snow-ballers that their intended activity did not fall within the hazy rubric of ‘reasonable’; elderly dog-walkers harassed by police drones. ‘Badly misjudged’, as one former Lord Chancellor observed, of the latter incident.^29^ Just a bit. Medieval shaming-rituals are only supposed to exist in history textbooks. In its report, the APPG warned of a ‘material’ diminution of ‘public confidence in policing’.^30^ Dickens’s Mr Bumble put it succinctly. The law should not look an ‘ass’.^31^

Which brings us to a couple more marketing disasters. Cautionary tales, to reinvest Bagehot’s metaphor, of sorcerers trapped in their own ‘rough magic’.^32^ First, Dominic Cummings and his bizarre trip to a land very ‘far, far away’ in May 2020.^33^ To Country Durham in fact, to the in-laws, in search of child-care. It may have been legal, just about, given that the ‘regulations’ in place provided an exemption for parents *in extremis*; though the subsequent jaunt to the bluebell fields of Barnard Castle was less easy to reconcile. And then, a year on, the Health Secretary, Matt Hancock, discovered groping an ‘aide’ in his office. Moral depravity is hardly a bar to a life of sorcery, as any devotee of the classic fairy-tale knows. But the context can vary, and the regulations in place required all sorcerers to remain at least two meters apart from any fair maiden they were trying to bewitch. A couple of days later, the lovelorn Secretary found that his magical powers had been ‘all o’erthrown’.^34^

As, of course, always happens. For no magic lasts for ever:

The charm dissolves apace;

And as the morning steals upon the night,

Melting the darkness, so their rising senses

Begin to chase the ignorant fumes that

Mantle their clearer reason.^35^

Time to move on, to our third ‘gathering’. Which, to retain Prospero’s parlance, ‘transports’ us to Bristol.

### A Weekend in Bristol

A couple of gatherings in fact. The first just a week after the Sarah Everard vigil, on 21st March. A series of ‘kill the bill’ events had taken place over the preceding days, held to protest against the government’s prospective *Police, Crime, Sentencing and Courts Act*. The passing irony is patent; a series of protests against attempts to inhibit protest. The events on the 21st followed a customary course. An initial request to hold a march turned down by the police, who asked people not to attend for reasons of ‘public health’. Some heeded, some did not. A peaceful gathering which became more agitated, an order read out, which no-one appears to have heard, and a decision to break it up by force, followed by a predictably frantic attempt to exculpate. An evening of ‘thuggery’, pure and simple, the Home Secretary declared the following day. Violent, certainly, but the situation was hardly simple. The fact situation was contested, the law far from clear, and plenty of other ‘kill the bill’ marches in other cities had passed off peacefully enough.^36^

In order to better understand what happened in Bristol on the 21st we need to think about causation, both in the immediate and the slightly longer-term. The immediate reason is found in the ‘bill’ that the marchers were hoping to ‘kill’, more particularly a number of provisions in Part 3, which amend the law in regard to ‘protests’. In effect, criminalising the existing law of public nuisance. Most obviously in Sections 55–57. Section 55 amends section 12 of the 1986 *Public Order Act*, to vest increased powers in the police to prohibit any ‘procession’ which might ‘result in serious disruption to the activities of an organisation which are carried out in the vicinity’ or may ‘have a significant and relevant impact on persons in the vicinity’. Section 55.4, notably, gives the Home Secretary power to determine, in secondary legislation, the meaning of ‘serious disruption’. Section 56 replicates the same for ‘static’ gatherings. Section 57 provides increased penalties for those convicted of holding, or joining protests, which have not been permitted by the police.

There is, of course, the customary declaration of compatibility with Convention rights, purposed to ward off potential legal challenges. The Convention right most obviously relevant being Article 11, the Freedom of Assembly and Association. The sub-clause of which permits ‘restrictions’ where ‘prescribed by law’ and ‘necessary’ in the ‘interests of national security or public safety, for the prevention of disorder or crime, or ‘for the protection of health or morals’. Whether this extends to merely annoying the neighbours, as section 55 might, is questionable. The reason why the government feels the need to place further restrictions on the right to protest, outwith present Covid regulations, can be found in the sentiment which underpins section 55. It is here that we need to conjure the slightly longer perspective.

More precisely the consequence of an earlier march in Bristol, on 7th June 2020. This march, which took place during a brief lull between lockdowns, was organised in support of the Black Lives Matter movement. And culminated in a series of indignities inflicted on the statue of an eighteenth-century slave-trader named Edward Colston, which had stood on the dock-side since 1895. The idea that something might be done about the statue had been ventured before; hardly a man, it was supposed, deserving of commemoration.^37^ But nothing had been done.^38^ And so Colston stayed, until the 7th, when his statue was dumped in the harbour. Not initially much regretted, at least not by the city Mayor who confessed that he ‘felt no sense of loss’. In contrast to the Home Secretary, who condemned the dunking as ‘utterly disgraceful’.^39^

A series of protests followed against more statues scattered about the country, to include not just slave-traders, but others who might be variously termed imperialist.^40^ And a line appears to have been crossed when a statute of Winston Churchill, standing in Whitehall, was graffiti-ed with the word ‘racist’, first during a parallel BLM march on the same day, and then again a few weeks later during an Extinction Rebellion parade.^41^ Sections of the press demanded something must be done. And so it was. Section 46 of the new Act amends Section 22 of the Magistrates Court Act, so that anyone found guilty of damaging a ‘memorial’ of ‘commemorative purpose’, to include surrounding flora and fauna, can be convicted of a specific criminal offence, and sentenced to up to 10 years in prison; a considerable uplift on the previous maximum of three months.^42^

The concerns are evident. Some are principled, of the kind which underpinned Lord Justice Laws’s renowned observation, on being asked to prohibit another annoying gathering a decade ago:

Rights worth having are unruly things. Demonstrations and protests are liable to be a nuisance. They are likely to be inconvenient or tiresome, at least perceived as such by others who are out of sympathy with them.^43^

Others assume, once again, the sillier aspect. A statute designed to protect statues. Milton would certainly have found it ridiculous. Better still to pass legislation requiring the ‘casting down’ of ‘idols’, the purpose of which was to keep the gullible ‘fatally stupifi’d and bewitch’d’.^44^ A witchery which found reference in the government’s briefing on the intended Act. The ‘emotional and wider distress’ that the damaging of statues might have amongst certain members of the public. We might, in similar vein, anticipate what Shelley might have thought. He did write a poem on the subject, *Ozymandias*, published in early 1818:

Half sunk, a shattered visage lies, whose frown,

And wrinkled lip, and sneer of cold command,

Tell that its sculptor well those passions read

Which yet survive, stamped on those lifeless things,

The hand that mocked them, and the heart that fed…^45^

Ozymandias is the cannibalised version of the Greek name for the Pharaoh Rameses II, a serious slaver.^46^ There is a prosaic interpretation of *Ozymandias*: time passes, and statues fall down. And a subtler. Statues are representative, and in the falling the representation mutates. We still comprehend them, but we do so differently. The statue of Edward Colston can still be seen on display in a city museum, replete with its new paint-work. Not left out of laziness, but because the colouring adds something to our appreciation of the statue, and its history. We might, or more likely not, be much offended by the dunking of Edward Colston. But we are certainly wiser for it.

Time will tell the extent to which the provisions in the new *Act*, which duly secured Parliamentary approval the following July, will inhibit protest. They could, though for reasons we will shortly consider, the inscription of so many ‘dodgy definitions’ will militate against effectiveness.^47^ In the meantime, though, we are left to reflect further on some discomforting ironies. Most obviously the patent inconsistencies in regard to the policing of different protests over the last year; something which is hardly likely to diminish as a consequence of the new *Act*.^48^ The fact that large scale marches in support of certain causes might be permitted, even applauded, whilst others, to include protests against a bill purposed to inhibit protests, are not.^49^ Or indeed vigils held in memory of murder victims. A degree of inconstancy is to be expected in the discretionary enforcement of any law, even outside a public health emergency. But there does come a point at which the inconsistency is so dramatic that it detracts from what Austin termed the ‘binding virtue’ of the law’.^50^ Something which invites us to consider a little more closely the jurisprudential consequence of writing ‘gibberish’.

## Gibberish

It should be beyond argument that clarity in the law is a virtue, for reason of both utility and justice. Especially, it might be supposed, where the law touches on what we might imagine to be ‘fundamental’ rights. Of the kind Lord Hoffmann had in mind, when he affirmed, in the plainest of terms, that such rights should never ‘be overridden by general or ambiguous words’.^51^ Having revisited these variously unsettling histories, past and present, our next task is to weigh the virtues of clarity against those various expressions of ‘lockdown-law’ which were purposed to suppress protest and gathering.

### Wild Waters

There was not much that Milton had in common with his near-contemporary Sir Francis Bacon. This though; the surmise that laws badly drafted may not be properly termed law at all. ‘So if the law give an uncertain sound, who shall prepare to obey it’, Bacon wondered, before surmising that ‘certainty is so essential to law, that law cannot even be just without it’.^52^ A logic which Austin would later, and famously, stretch in his *Province of Jurisprudence Determined*, published in 1832. In Austin’s opinion, clarity of exposition was critical in the writing of good law.^53^ The ‘term laws’ can be made ‘extremely ambiguous’, sometimes because it is ‘improperly applied to various objects which have nothing of the imperative character’, or because it is derived form ‘muddy speculations’ or founded on ‘mysterious or imposing attributes’ or ‘imposed by general opinion’.^54^ Or for the simple reason that the supposed law is obscured by ‘nonsense or jargon’.^55^ All of which was of the greatest importance for the simplest of reasons. Muddy law militates against comprehension and, as a consequence, the sustainability of the ‘habit of obedience’.^56^

A good point at which to turn our closer attention to ‘lockdown-law’. The framework for which is found in three statutes. Two are pre-existing, the 1984 *Public Health Act* and the 2004 *Civil Contingencies Act*. Neither were purposed for a viral pandemic. The latter was intended to deal with terrorists, but included useful clauses which facilitated the detention of anyone who seeks to disrupt ‘services relating to health’, or otherwise threaten ‘damage to human welfare’.^57^ The former assumes greater import, because it was from this Act that the mass of secondary legislation, which would inscribe ‘lockdown-law’, assumed its legitimacy. The 1984 Act provides a range of broader discretionary powers in for use a public health emergency, the most serviceable of which are found in section 45 C(4)(d), which permits the Secretary of State to exercise any ‘special restriction or requirement’. The section expressly makes mention of ‘events or gatherings’, but does not define either.

The *Coronavirus Act* is a vast document, in its original form comprising 102 sections and 29 schedules, many of which were purposed to bolster public health services. The greater controversy moves around the legality of Schedule 21, which permits the detention of ‘potentially infections persons’, Schedule 22 which can be used to prohibit ‘mass gatherings’, and Schedule 23 which permits a wide range of surveillance powers. Here again, there is no definition of ‘mass gatherings’. Given that the purpose of the *Coronavirus Act* was to provide a bit of cover, the masque of parliamentary process, the absence of much by way of definition is hardly surprising. Where there might be a meaningful distinction, between the ‘gatherings’ written into Schedule 22 and those alluded to in subsection C(4)(d) of the 1984 Act, is the possible application of the former to non-infected persons. Though we can only surmise; as we can the countervailing assumption that the meaning of the latter is for the ‘restriction’ on the movement of the infected only.

The substance of ‘lockdown-law’ is, of course, discovered in the hundreds of provisions published in successive *Health Protection *(*Coronavirus, Restrictions*)* Regulations*, under the *aegis* of section 45. Two concerning presumptions to note, in passing; subsection C, the predictable self-certification of Convention compatibility, and subsection R, which, ‘by reason of urgency’, justifies the limited presentation of successively amended regulations for Parliamentary scrutiny. We will revisit the issue of legislative scrutiny in due course. Our immediate concern is with the consequential mess. Evident, perhaps most obviously, in the confusion regarding the generic difference between legally-enforceable rules and statements of mere ‘guidance’. So evident, indeed, that the conspiratorially-inclined have surmised that the confusion might be ascribed to what Shakespeare termed the raising of ‘wild waters’.^58^ Deliberate, in other words.

Various social distancing ‘rules’ fall into the category of ‘guidance’, as does the instruction, in certain circumstances, to self-isolate after coming ‘in contact’ with an infected person.^59^ None of which has ever been, in strict terms, legally enforceable. The same is true of much ‘operational guidance’ published by the Education Department for school ‘leaders’, including ‘bubbles’ and contact tracing. In regard to the distinction between legal and non-legal obligations, the government’s Coronavirus ‘legislation’ website says simply this, ‘Guidance and advice is likely to be based on legislation (in which case it will be legally binding)’. To which is then added the observation that guidance ‘might be a mixture of what you must and what you should do’.^60^ The ‘is’ and the ‘ought’ of lockdown-law; gibberish in its purest form.

Our closer concern is, though, with the attempt to constrain smaller gatherings during the pandemic. Something which has its own, predictably confusing, history. For the present we only need to note the first part of this history, from March 2020 to September. By way of a timeline; all gatherings, of more than two, were prohibited during the first national lockdown, announced in 23 March; on 1st June this was adjusted to the so-called ‘rule of six’, at which point a first definition of ‘gathering’ was written into the regulations. A ‘gathering’, it was suggested, comprised ‘two more persons… present together in the same place’, for the purposes of ‘social interaction’.^61^ Not that there was a definition of ‘same place’, nor indeed ‘present together’. And an odd invocation, it might be thought, to run alongside a ‘rule of six’. Odder still when, on 4th July, the number was raised to thirty, in the event of an enumerated ‘gathering’.^62^ And then, on 15th September, it was back to the ‘rule of six’, courtesy of the *Health* *Protection* (*Coronavirus*,* Restrictions*) (*No* *2*) (*England*) (*Amendment*) (*no 4*)* Regulations*.^63^ At which point we will pause to consider what might, amidst some pretty stiff competition, be judged the most gibbered piece of legal drafting of the entire Covid-experience. That which sought to recreate a law against ‘mingling’.

### The Law of Mingling

The re-imposition of the ‘rule of six’ was announced at 1140 on the evening of the 14th September, to come into force twenty minutes later. Here again some might detect a darker sorcery, the raising of ‘wild waters’. Conversely, it might just be a consequence of gibberish thinking leading to gibberish law. The scattergun nature with which so many regulations were announced during the pandemic intimates the latter. As did the rather frenetic appearance of the Home Secretary the following day, venturing onto the airwaves to try to explain what was going on. Before we see what she had to say, we should acquaint ourselves with the relevant piece of secondary legislation.

Sub-sub-sub-sub section 2(3)(2B)(b)(ii) of amended Regulation 5 reads that no-one should ‘otherwise mingle with any person who is participating in the gathering but is not a member of the same qualifying group as them’. Founded on the broader proscription of ‘gatherings’ discovered in 5(5G), which was re-defined as occurring where ‘two or more people are present together in the same place in order to engage in any form of social interaction with each other, or to undertake any activity with each other’. All of which leaves the interested reader to wonder what a ‘social interaction’ might be, and indeed an ‘activity’. It is here that the ‘exceptions’ could help; or not. Amongst the explicit was a ‘gathering’ in a household of more than six, certain work and education exceptions, weddings, funerals and other ‘significant events’, along with various sporting and ‘licensed’ activities, to include grouse-shooting and paint-balling.^64^ Everything else was left to the discretion of policing authorities. Interestingly, the exceptions made specific reference to ‘protests’, so long as they were organised by a ‘business, a charitable, benevolent of philanthropic institution, a public body, or a political body’, and provided the organiser could persuade the police that they had undertaken a satisfactory risk assessment. Something which, as we have already noted, creates an expectation that the police must engage reasonably in the process, and not consider the ‘hands’ to be simply ‘tied’ by the regulations.

It was evident, though, that ‘mingling’ was meant to be rather different. Sadly, though that difference was not itself evident. No-one knew what it meant. It was wondered if the placing of the ‘mingling’ provision imputed application only where there might be a nearby sanctioned ‘gathering’ to which the prospective mingler had not been invited. Maybe, maybe not. Otherwise, there was simply the discretion of passing police officers, and a bottomless pit of prospective anomalies, commonly of the ridiculous variety. A casual chat over a garden-fence perhaps, three on one side, four on the other. Did it make a difference if they were stood or moving up and down the fence? We might recall that it was the ‘static’ nature of the ‘gathering’ at Clapham Common which led to the band-stand assault. We do not need to conjure more examples to appreciate that the law of mingling was a classic piece of ‘gibberish’. One incredulous MP was led to wonder, tongue at least partly in cheek, whether there was now a difference in law between mingling, ambling and merely pottering around.

And all despite the best efforts of the Home Secretary, who was invited to lend some clarity the following day in a BBC studio. She might as well have taken a seat at the Mad Hatter’s picnic-table. Mingling, she ventured, should be understood as ‘people coming together’; which came close to the idea of being ‘present together’, though perhaps not quite as close as she thought, one rather obviously more ‘static’ than the other. She was, though, unable to extrapolate further, at least on the semantics. And so, instead, sought sanctuary in numbers, taking the opportunity to explain what six meant. Which was somewhere between five and seven, and certainly less than eight; the latter of which could be comprised of two groups of four, and which was ‘absolutely mingling’. At which point, having shed as much light as she could, the retired, back down the rabbit-hole. With the parting shot that the situation would be helped if everyone could keep a close eye on their neighbours, to see that they were compliant. She certainly intended to.

Oddly, the police were nothing like so convinced that the ‘war on the virus’ might be best defeated by an army of ‘curtain-twitchers’. There was not the resource to send officers chasing around the streets on the behest of twitchers. Neither, more importantly perhaps, could they make much sense of the new provision. Or most of the older. They were still asking for further clarification in regard the still relatively recent amendment which granted them discretionary powers to break-up larger ‘gatherings’ of thirty or more. How did that stand in light of the new provisions introduced on 15th September? The situation was not, in the context much helped, by the Home Secretary’s collateral advice, that the new law against mingling should understood in the ‘context’ of ‘keeping our distance, wearing masks’. The latter had some legal force, in certain circumstances, the former none. It was simply ‘guidance’. Whether the Home Secretary herself knew the difference is matter for conjecture.^65^ Hardly surprising if there was a sense of weary despair in the Federation chair’s observation, that ‘trying to interpret’ the morass of shifting, ‘rules’ was proving well-nigh impossible. Police officers were as ‘confused’ as the public, and the intended law of mingling only made things worse.^66^ The chair of the Metropolitan Police Federation expressed similar dismay. Unless something was done about the mess, and quickly, there would be a ‘perfect storm’.^67^

A prophesy that would find a tragic prescience six months on. By which time, the aligned histories of ‘gathering’ and ‘mingling’ had mutated further, generally in response to a bewildering array of different metaphorical strategies designed to persuade everyone to ‘stay at home’; tierings, traffic-light systems, mole-whacking. On 5th November, a second national lockdown reduced the number allowed to gather back to two. On 2nd December it was back up to six. On 20th December it was back down to two, for anyone living in a Tier 4 area. On 5th January, everyone found themselves in Tier 4; which is when snowball-fighting was criminalised. It was also the regime in place when Sarah Everard was murdered. There might be a reasonable excuse as to why the law kept changing so frequently, and so suddenly. The inability to provide either the police or the public with sufficient notice or ‘clarity’ is less excusable. As the Parliamentary Joint Committee on Human Rights concluded. It was, in their considered opinion, the lack of ‘clarity’ which led directly to the shameful scenes witnessed at Clapham Common on 13th March.^68^

## The Crisis of Parliamentary Democracy

It remains to conjure some conclusions, past and prospective. We will start in the past, with the publication in 1921 of a provocative essay entitled *The Crisis of Parliamentary Democracy*.^69^ It ventured the possibility that the democratic institutions of the fledgling Weimar Republic were so weak that they would be unable to withstand concerted pressure from non-democratic political forces. It might be thought a distressing resonance, given the future history of the Republic, and indeed the future history of the essay’s author, Carl Schmitt. How sharp the resonance will be for coming generations of historians to decide. It might seem overblown, or darkly prescient. As we wait to see, we might contemplate two of Schmitt’s collateral observations. That an indeterminate ‘state of exception’ is a species of dictatorship. He categorised it as ‘commissarial’. And that it is facilitated by the failure, on the part of the legislative and the judiciary, to hold government to effective account. The surest sign, he observed, of a democracy in distress.^70^

Something to ponder as we reflect back on the passing ‘age of Covid’. We can acknowledge the presence of parliamentary scrutiny, and occasional legal challenges against various aspects of ‘lockdown-law’, without accepting that they either suffice or convince.^71^ We can wonder when an Appeal Court concludes that a ‘lockdown’ backed by swingeing criminal sanctions does not represent a ‘deprivation of liberty’ as understood in Article 5 of the Convention; not least by wondering when, then, it might.^72^ And indeed when it supposes that the right to protest written into Article 11 of the Convention can be suspended in the ‘public interest’, where the regulations provide a notional ability to gather where there is ‘reasonable excuse’ to do so.^73^ But then, when there is an evidently ‘reasonable excuse’, as in the Everard vigil, there seems to be little that can be done if senior police officers fail to properly comprehend their legal obligations.

It is this latter ‘right’, to protest, which is our closer concern. A right which is often assumed to be a litmus-test of a credible parliamentary democracy.^74^ We do not need to reconcile ourselves to Schmitt in order to appreciate that its suspension might be an especially concerning sign of a distressed democracy. Our own history, as Lord Denning famously supposed, is ‘full of warnings against the suppression of the right to protest’.^75^ The ‘Peterloo massacre’ is just one of the more dramatic. We might go back rather further, to Milton again. And his renowned essay in defence of free expression, *Areopagitica*, published in 1644. There is no clearer evidence of tyranny than the attempt to suppress common ‘grievances’, and no more futile an endeavour. He famously likened it to the ‘gallant man who thought to pound up the crows by shutting his park-gate’.^76^

And then forward again, to 1859, and another defining texts in the history of liberal philosophy, John Stuart Mill’s *On Liberty*. At the heart of which can be found the critical difference, between mechanisms of ‘legal coercion’ and those of mere ‘persuasion’. The former being reserved only for actions ‘calculated to produce evil to someone else’.^77^ For the rest, there is what we might term ‘guidance’. Needless to say, high on Mill’s list of cherished liberties is that of free expression, for ‘only through the diversity of opinion’ is there ‘a chance of fair play to all sides of the truth’.^78^ Followed shortly after, by the freedom to decide, to embrace the ‘contingencies’ of life, to calibrate risks and to act accordingly.^79^ It comes as no surprise to discover that, in fashioning his ‘harm principle’, Mill took inspiration from Shelley’s *Ode to Liberty*.^80^ Hope and Liberty; ‘treasures to be bought/By blood or tears’ (268–269).

We might, as we move to a conclusion, return to autumn 1819. Shelley was certainly not alone in his condemnation of the events which had taken place at St Peter’s Field. A young journalist named John Tyas, who had journeyed to Manchester, filed a damning report in the *London Times*, wondering, not least, if those gathered really had represented an ‘unlawful assembly’, that warranted the reading of the Riot Act. Or whether it was just an excuse to disperse a ‘gathering’ that was bothering some business ‘carried out in the vicinity’. Another journalist present was John Edward Taylor, who immediately began a subscription to found another newspaper. The first edition of the *Manchester Guardian* appeared in spring 1821, advertising in its prospectus that it would ‘zealously enforce the principles of civil and religious Liberty’.

Not that Sidmouth seemed inclined to compromise. Cavilling journalists and activist poets were not going to deter him from doing his duty. Noting Tyas’s comments, he immediately set about strengthening the statutory regime. A couple of months later, Parliament passed a *Seditious Meetings Act*.^81^ It required any who wished to convene a meeting of more than fifty, for the purpose of questioning matters of ‘church and state’, to first get the permission of the sheriff or a local magistrate. To which was added a further proviso that prevented the attendance of anyone from outside the parish. Early-day tiering, to suppress the virus of complaint, and stop folk wandering about. There would be no more insolent mingling on Sidmouth’s watch. History, as Mark Twain is said to have said, does not always repeat; but it commonly rhymes.

There will, in summer 2022, be a public inquiry into governmental responses to the coronavirus pandemic. It might invite along a few historians, and indeed poets, to contemplate the resonances. But probably not. More likely preoccupied by the prosaic question of why, despite an array of natural advantages, the UK ended up with the highest aggregate death rate in Europe, at the greatest economic cost.^82^ After which there will be a temptation to consign ‘lockdown’ to history; a passing visit to dystopia. It is here, though, that we should be careful. Because Covid-19 will not be the last pandemic, certainly not the last supposed threat to the ‘health of the nation’. History proves that. For which reason the successive lockdowns of 2020–2021 will not be the last either.

Whether that leads us to share Lord Sumption’s misgivings is a matter for individual contemplation. The most ‘coercive’ regulatory regime ever ‘imposed’ by the ‘state, a ‘monument of collective hysteria and government folly’. A ‘remarkable… departure from our liberal traditions’, effected with a ‘cavalier disregard’ for the principle of the rule of law.^83^ An opinion which should surprise no-one who has read the foreboding conclusion to his *Law in a Time of Crisis*:

We will not recognise the end of democracy when it comes. Advanced democracies are not overthrown… Instead, their institutions are imperceptibly drained of everything that once made them democratic. The labels will still be there, but will no longer describe the contents. The façade will still stand, but there will be nothing left behind. The rhetoric of democracy will be unchanged, but it will be meaningless. And the fault will be ours.^84^

It is tempting to end here. But Shelley would have us hope, if cautiously, and he closed his *Mask* in this register:

Stand ye calm and resolute…

With folded arms and steady eyes,

And little fear, and less surprise,

Look upon them as they did slay

Till their rage has died away. (319, 344-7)

Three years later, Shelley was dead, leaving behind an unfinished poem entitled *The Triumph of Life*. A metaphysical sequel to the *Mask*. No less hopeful, that ‘surging humanity’ will sweep aside the ‘hoary anarchs, demagogues, and sage’, but reconciled also to the ‘rhyme’ of history, which intimates that struggle between Anarchy and Liberty is protracted and painful (237). Shelley knew that the ‘Peterloo massacre’ would not be the last. Nothing that happened at Clapham Common on 13th March, or in Bristol a week later, or indeed six months earlier, would have surprised him. Nor should it us.

## Footnotes

1 J. Milton, *The Tenure of Kings and Magistrates*, in *Areopagitica and Other Writings*, (Penguin, 2014), 144. The ‘men’ Milton had more immediately in mind were the Presbyterian divines and MPs who opposed the trial of King Charles I.

2 Not quite novel. The 1393 *Cloth Act*, 11 Edw.3.c2, proscribed a different kind of ‘mingling’, of English with continental textiles.

3 The idea that the coronavirus pandemic was both unprecedented and unpredictable has been furiously spun. Nonsense on both counts. As to the unprecedented, history is full of pandemics. The Old Testament is littered with them; ten plagues in *Exodus* alone. Whilst our medieval and early modern ancestors found a year without a plague odder. As for viral pandemics of more recent vintage, the ‘Russian’ of the 1890s will ring a bell with contract lawyers. It was the ‘Russian’ flu that Mrs Carlill caught. The largest, in terms of fatalities, was, of course, the ‘Spanish’ flu of 1918–1920, which claimed somewhere between 20 and 50 million victims. To be followed, later in the century, by the ‘Asian’ and the ‘Hong Kong’ flu epidemics, together with various other global pandemics, including tuberculosis, polio, and HIV. There is nothing surprising, or unprecedented, about a pandemic. Moreover, Covid-19 is the third coronavirus to reach pan or epidemic proportions this century, following MERS and SARS. See K. Sahu, A. Mishra and A. Lal, ‘Coronavirus disease 2019: an update on the third coronavirus outbreak of the twenty-first century’, 113 *QJM* 2020 384–6. On the ‘inevitability’ of a pandemic, prophesied in the years leading up to Covid-19, see I. Morris, ‘Plagues and Socioeconomic Collapse’ and L. Borysiewicz, ‘Plagues and Medicine’, both in J. Heeney and S. Friedemann (eds), *Plagues*, (Cambridge UP, 2017), at 85 and 154 respectively. As for the attendant myth of the unpredictable, the likelihood of a viral pandemic was gamed just a few years ago. Exercise Cygnus, designed to test the resilience of the NHS. Barely, was the prophetic conclusion. A similar exercise was conducted, at much the same time in the US, at the Johns Hopkins Center for Health Security. The results no less sobering.

4 W. Shakespeare, *The Tempest*, (Routledge Arden, 1964), 5.1.50.

5 He was presently struggling with redrafts of his verse-drama *The Cenci*.

6 A number of early-day Women’s Suffrage banners too. The reporter Samuel Bamford noted that the stage upon which Hunt was to speak was ringed by some of the ‘handsomest girls’. Compelling, if very different, accounts of the Peterloo ‘massacre’ can be found in E. Thompson, *The Making of the English Working Class*, (Penguin, 1991), 734–68, and J. Chandler, *England in 1819: the Politics of Literary Culture and the Case of Romantic Historicism*, (Chicago UP, 1998), 15–21.

7 The precise number of consequential deaths, in the longer-term, is unknown. The official tally was eleven dead and 421 injured, 162 with sabre-wounds.

8 See R. Holmes, *Shelley: the Pursuit*, (HarperCollins, 1994), 329–30.

9 Composed over what his biographer Richard Holmes terms the ‘most creative eight weeks of his whole life’. See Holmes, *Shelley*, 532.

10 Beset by myriad Chancery disputes regarding estate succession and child custody, Shelley harboured a peculiar animus towards the Lord Chancellor. ‘Thy country’s curse’, as he put in in the opening line of his *To the Lord Chancellor*.

11 All internal references from P. Shelley, *Complete Poetical Works*, (Oxford UP, 1971).

12 ‘The master theme of the epoch in which we live’, as he confirmed in correspondence with Byron. See Holmes, *Shelley*, 346.

13 Holmes, *Shelley*, 261.

14 In the early 1790s, Godwin had been at the forefront of English Jacobinism. He was also Shelley’s father-in-law. Mary Godwin was Shelley’s second wife, and author of *Frankenstein*. For Godwin’s influence on Shelley, see Chandler, *1819*, 28–9.

15 Holmes, *Shelley*, 531.

16 The masque is most commonly associated with the early Stuart Court. Charles I was a particular enthuse iast. For an account see N.Maguire, ‘The Theatrical Mask/Masque of Politics: The Case of Charles I’, 28 *Journal of British Studies* 1989, 1–22.

17 Hunt sought to ameliorate a difficult situation by supposing that Shelley’s prospective ‘public had not become sufficiently discerning to do justice to the sincerity and kind-heartedness of the spirit that walked in this flaming robe of verse’. In *Works*, 345.

18 The right moment, it transpired, was 1832. The *Mask* was finally published amidst the agitation which accompanied the passage of the ‘great’ Reform Act.

19 See evidence taken by the All-Party Parliamentary Group, in its report *Police Powers and the Right to Peaceful Protest*, published 1 July 2021, with the Institute for Constitutional and Democratic Research. Paras.119–23.

20 *Leigh *et al. *v Commissioner of the Metropolitan Police and the Secretary of State for Health* [2021] EWHC 661 (Admin). The court had been asked to grant interim declaratory relief. The judge declined to grant the remedy, but emphasised that there was nothing in the regulations which made such a ‘gathering’ unlawful, further noting that the police had earlier intimated that agreement should be possible. Only then to claim that there ‘hands were tied’ by the regulations; which was wrong in law. The regulations, as we will see, expressly leave open the possibility of a ‘gathering’ if there is ‘reasonable excuse’ and an accommodation can be reached.

21 APPG report, para.6.

22 In *Works*, at 345.

23 Whilst she desperately tried to distance herself from the fallout, a Home Office source confirming that the ‘decision to start pining women to the floor and dragging them away from the Clapham Common bandstand was an independent operational decision’. Reports posted on the *Guardian* and the *Independent* websites on 15th March.

24 W. Bagehot, *The English Constitution*, (Cambridge UP, 2001), 4, 8–9. The term ‘cheapjack’ was coined for Disraeli, first used in *Punch*. Bagehot loathed Disraeli. See S. Weintraub, *Disraeli: a biography*, (Hamish Hamilton, 1993), 273, 450–1.

25 The greatest of constitutional ‘anomalies’. See Bagehot, *Constitution*, 30–1, 49.

26 Dismissed, with little evident regret, in the opening paragraphs of the first chapter of the *English Constitution*.

27 Speaking more closely to the role of monarchy, but the metaphor is of broader pertinence. See Bagehot, For a recent, and intriguing, account of modern government as a mutant species of the ‘occult’, see P. Monateri, *Dominus Mundi: Political Sublime and the World Order*, (Hart, 2018), chapters 3 and 4.

28 J. Austin, *The Province of Jurisprudence Determined*, (Cambridge UP, 1995), 166.

29 Comments of David Gauke, reported by the BBC 27/3/20. The supermarket-trolling Chief Constable was located in Northamptonshire. It was the Much Wenlock division of the West Mercia Police which expressed alarm at prospective snow-ballers taking to the streets and hurling viruses around. The elderly dog-walkers were spotted in the Peaks, courtesy of a drone despatched by the Derbyshire Constabulary.

30 For a commentary on the undoubted challenges faced by the police during the pandemic, see D. Sheldon, ‘Policing the Pandemic: Compliance and Legitimacy during Covid-19’, 32 *Kings Law Journal* 2021, 14–25.

31 C. Dickens, *Oliver Twist*, (Penguin, 2002), 436.

32 Shakespeare, *Tempest*, 5.1.50. The best way to defeat a sorcerer, as any Harry Potter fan will know; the art of ‘transfiguration’. Or indeed any scholar of the medieval occult; the so-called ‘mirror’ trick by which the sorcerer is trapped in his or her own reflection.

33 A referent more resonant for all of us with recent child-care experience. From the film series *Shrek*. Full of wizards and monsters, evil counsellors and laws that make no sense.

34 Shakespeare, *Tempest*, Epil.1. Other sorcerers had fallen similarly, assorted modellers and medics included. Amongst the earlier, and in its moment spectacular, was the entrapment of Professor Neil Ferguson, head of the team of modellers based at Imperial College whose figures had freaked the Prime Minister in March 2020. Committing much the same sin, against Covid-regulations at least, as the Health Secretary. Groping when he should have been isolating. Slightly different in the case of Scotland’s chief medical officer, who was caught in April 2020 journeying to a second home in contravention of existing rules, repeatedly.

35 Shakespeare, *The Tempest*, 5.1.64–8.

36 All noted in the APPG report, which also looked at the policing of the Bristol ‘gathering’. The suspicion that not all might have been what it seemed was reinforced by the subsequent retraction by the Avon and Somerset Police that a number of officers had suffered serious physical injuries, including fractured legs and ribs. Later retracted. There had, the statement concluded, been ‘no intention to mislead’.

37 Colston, who died in 1721, was a prominent member of the Royal Africa Company, which is thought to have traded around 84,000 African slaves. For much of the eighteenth and nineteenth-centuries, Colston was praised for his philanthropy in the city, founding schools and almshouses and so on; with no-one pausing to contemplate for too long where he had gotten the money.

38 There had been agreement in 2018 to erect a plaque at the statue describing Colston’s past activities; but it had then been vetoed by the city Mayor.

39 *Guardian* report, posted online, dated 22nd March 2021.

40 The protest which seemed to gain most traction, in the press at least was directed against a statue of Cecil Rhodes, perched on the side of Oriel College in Oxford. After initially intimated that it might remove the statue, the College changed its mind; for reason of planning ‘complexities’. Later, in December, the statue of another slave-trader, Robert Milligan, was removed from Canary Wharf.

41 The press, oscillating between sentiments of disgust and titillation, was especially exercised by the fact that a number of female protestors who had chained themselves to Winston had been ‘topless’. See ‘Churchill statue vandalised again as topless climate protestors chain themselves to railings’, Sky News report 11th September 2020.

42 By means of taking away the existing provision which required that anyone who is deemed to have inflicted damage of less than £5000 value could only be sentenced summarily to a maximum of 3 month’s imprisonment and/or a fine of £2500.

43 In *Tabernacle v Secretary of State for Defence* [2009] EWCA civ 23. The ‘gathering’ here was a protest-camp established at Aldermaston Atomic Weapons Establishment.

44 J. Milton, *Eikonoclastes*, in M. Hughes (ed) *Complete Prose Works of John Milton*, (Yale UP, 1953–82), 3.346–7. For a commentary on Milton and the ‘casting down’ of icons, see L. McKnight, ‘Crucifixion or apocalypse? Refiguring the *Eikon Basilike*’, in D.Hamilton and R. Stier (eds) *Religion, Literature, and Politics in Post-Reformation England 1540–1688*, (Cambridge UP, 1996), 149–54.

45 Lines 4–8, in Shelley, *Works*, at 550.

46 Whose statue had recently been acquired by the British Museum. Or at least the top half, nicked from his mortuary temple at Thebes by an Italian named Giovanni Belzoni. Belzoni specialised in raiding mortuary temples, and then selling on his ill-gotten gains to gullible aristocrats or, at a variant, gullible museums. The statue in question, was taken from the temple in 1816, acquired in 1818 and finally went on show in 1821.

47 As Sir Peter Fahy, former Chief Constable of Manchester, put it. Quoted in *Liberty’s Briefing on the Police, Crime, Sentencing and Courts Bill for Report Stage in the House of Commons*, July 2021, at 10. The same view reached in the APPG report, at paras.161 and 171.

48 A point emphasised, repeatedly, in the APPG report. See paras.161, 171.

49 Not just inconsistencies represented by BLM or Extinction Rebellion protests, but also football celebrations. A series of such gatherings in Glasgow have attracted a certain notoriety. An estimated 4000 Glasgow Rangers FC supporters gathered in George Square in Glasgow on 7th March, just a week before the Everard vigil, to celebrate winning the Scottish premiership. On this occasion there was a small number of arrests, for ‘public order’ offences, but none for any breaches of Covid ‘restrictions’. A similar number would gather again on 14th May, for the final home game of the season, watched by two dozen police officers. A greater police presence would not, a senior officer observed, been ‘proportionate for our resources’. For a comment on inconsistencies in policing protests, specifically noting the Glasgow ‘gatherings’, see the APPG report at para.160.

50 Austin, *Province*, 29–30, 285–6.

51 *Ex parte Simms*, [2000] 2 AC 131.

52 From his *Treatise on Universal Justice or the Fountains of Equity*, quoted in D. Coquillette, *Francis Bacon*, (Edinburgh UP, 1992), 244.

53 For a commentary on the importance of clear exposition in Austin’s jurisprudence, see W. Rumble, *Doing Austin Justice: The Reception of John Austin’s Philosophy of Law in Nineteenth-Century England*, (Continuum, 2005), 118–19, and also T. Bowser and J. McQuade, ‘Austin’s Intentions: A Critical Reconstruction of his Concept of Legal Science’, 29 *Campbell LR* 2006, 52–3, suggesting that the impulse might be attributed to Austin’s early experience as an equity drafter.

54 Austin, *Province*, 20, 36, 109, 123–124, 134.

55 Austin, *Province*, 31–32, 107.

56 Austin, *Province*, 166.

57 Part 1, section 1.2.h.

58 Shakespeare, *The Tempest*, 1.2.2.

59 Being ‘pinged’, in the vernacular, meaning notification by mobile phone ‘app’. Specifically exempted in the regulation.

60 Found under ‘Coronavirus’, on legislation.gov.uk.

61 Regulation 2, clause 6e. SI 2020/1374.

62 As listed in paragraph 6 of Schedule 3A, ‘a business, a charitable, benevolent or philanthropic institution, a public body or a political body’.

63 Inserted into SI 2020/684, by SI 2020/988.

64 It was reported that Cabinet has sat late into the night, earlier in the year, pondering how grouse-shooting might still be permissible; its thoughts tempered by the fact that the ‘second wave’ of the virus has arrived, very inconveniently, just as the shooting season was about to move into full swing.

65 The frequency with which the Home Secretary in particular seemed confused by the distinction between social distancing ‘guidance’ and the law was noted by the Parliamentary Joint Committee on Human Rights in its report entitled *The Government’s response to Covid-19: freedom of assembly and the right to protest*, 19th March 2021, para.59.

66 John Apter, quoted in T.Thompson, ‘NPCC to draw up guidance on ‘mingling’ to help officer police the rule of six’, in *Police Professional*, 14th September 2020.

67 Quoted in R.Camber, ‘Shop thy Neighbour’, *Daily Mail* headline, 14th September 2020.

68 JCHR report of 19th March, paras.59, 65.

69 C. Schmitt, *The Crisis of Parliamentary Democracy*, (MIT Press, 1988).

70 C. Schmitt, *Political Theology*, (MIT Press, 1985), 5. The ‘state of exception’ thesis has been reworked, somewhat controversially, by Giorgio Agamben, most obviously in his *State of Exception*, (Chicago UP, 2005), discussing Schmitt’s idea of ‘commissarial dictatorship’ at 33–36. For a commentary on Agamben and the Covid ‘experience’, see I.Ward, ‘The Law of Bare Life’, 72 *Northern Ireland Legal Quarterly* 2021, 26–51.

71 Parliamentary scrutiny taking the form of a review of coronavirus legislation every six months, and review of secondary legislation by the Delegated Legislation Committee each twenty-eight days.

72 This is not to ignore the familiar fact that all Convention rights are ‘qualified’. It is though to suppose that the qualification must be qualified too. If not, the right is barely worth the paper.

73 *R (Dolan) v Secretary of State for Health and Secretary of State for Education* [2020] EWCA Civ 1605, discussing Article 11 at Paras.103–105. The Court was persuaded by the presence of the ‘express possibility of an exception’, which meant that the broader restriction was not necessarily ‘disproportionate’. Leaving the legal philosopher to play the customary game of balancing rights, and the possibility that the right in Article 11 might be countermanded by the Article 2 ‘right to life’. For a comment on the dangers of dropping down this rabbit-hole, see the JCHR report, para.55.

74 One of the ‘fundamental’ rights of citizens in a ‘democracy’, according to Lord Hutton, in *DPP v Jones and Lloyd* [1999] 2 AC 286.

75 In *Hubbard v Pitt* [1976] QB 142 (AC).

76 J. Milton, *Areopagitica*, in *Areopagitica and Other Writings*, (Penguin, 2014), 113.

77 J. Mill, *On Liberty*, (Penguin, 1985), 68–69, and also 144. For the ambiguities written into the famed ‘principle’, see R.Reeves, *John Stuart Mill: Victorian Firebrand*, (Atlantic Books, 2008), 265–8.

78 Mill, *On Liberty*, 111.

79 Mill, *On Liberty*, 71–72, 75.

80 It has been surmised that Mill came to admire Shelley more the older he got. A youthful appreciation of the value of poetry in tempering the Benthamite ‘calculus’, further refined by his wife, Harriet Taylor Mill, who was every bit as admiring of Shelley. See J. Mill, *Autobiography*, (Penguin, 1989), 146, and Reeves, *Mill*, 66, 469–70.

81 One of the so-called ‘Six Acts’ that were passed in the closing months of 1819, each written in response to the summer of radical unrest.

82 The advantages being geographical and temporal; an island nation, granted significant advance warning of what was about to happen. Precise death-tolls will never be known, not least because the method of calculation is so crude. The aggregate death-toll for Covid-19 in the UK hovers around 127,000. The highest in Europe. At an estimated cost of around £215bn, as calculated at the first year anniversary of lockdown by the Centre for Economics and Business Research; a figure largely derived from Treasury estimates. It is, again, the highest figure in Europe. The extent to which the UK appears to have suffered a disproportionate financial hit is underlined by a series of Office for National Statistics reports which confirm that the contraction in its GDP, commonly assumed to be the more reliable indicator, has been the highest amongst G7 members for each of the four quarters leading up to March 2021. UK GDP is currently 8.7% down on pre-Covid levels, nearly double that of the next worst affected member, Canada.

83 J.Sumption, *Law in a Time of Crisis*, (Profile, 2021), 218–19, 225.

84 J.Sumption, *Trials of the State: Law and the Decline of Politics*, (Profile, 2019), 112.

